# Prevalence of Urinary Incontinence and Overactive Bladder Among Female University Students in Kazakhstan

**DOI:** 10.3390/epidemiologia6030038

**Published:** 2025-07-15

**Authors:** Yerzhan Sharapatov, Aluadin Nurberdiev, Nurbol Keulimzhayev, Aigul Botabayeva, Medet Toleubayev, Mariya Dmitriyeva, Rano Zhankina

**Affiliations:** 1 Department of Urology and Andrology, NJSC Astana Medical University, Astana 01000, Kazakhstan; erzhan.uro@gmail.com (Y.S.); nurberdiev.a@amu.kz (A.N.); nurbol_705.90@mail.ru (N.K.); aigi_05@mail.ru (A.B.); 2 Department of Surgery, NJSC Astana Medical University, Astana 01000, Kazakhstan; toleubayev.m@amu.kz (M.T.); dmitriyeva.m@amu.kz (M.D.)

**Keywords:** urinary incontinence, overactive bladder, cross-sectional study, young women, female students, risk factors, Kazakhstan

## Abstract

Background/Objectives: The aim of this research is to study the prevalence of urinary incontinence (UI) and overactive bladder (OAB) among female university students in Kazakhstan and to analyze the risk factors associated with these conditions. Methods: A total of 574 female university students aged 18 to 40 years participated in this cross-sectional, questionnaire-based study. Self-completed questionnaires including the International Consultation on Urinary Incontinence Questionnaire Short Form (ICIQ-UI-SF), the V8 Overactive Bladder Questionnaire (OAB-V8), and data on demographic and general characteristics of the participants were collected. The frequency, type, and severity of UI, as well as factors associated with these conditions were analyzed. Results: The median age of the participants was 22.00 years (IQR 19.00–24.00). The prevalence of UI among female students was 27.5% *(n* = 158). The mean ICIQ-SF questionnaire score was 1.00 (IQR 1.00–2.00), while the OAB-V8 questionnaire score was 2.00 (IQR 0.00–4.00). In addition, 10.1% of the respondents reported having OAB. The analysis showed that factors such as childbirth and physical activity level were associated with the presence of UI. In addition, childbirth, the presence of chronic diseases, and physical activity level were associated with OAB. The probability of UI increased by 2.3 times for women who gave birth (OR 2.30, 95% CI 1.16–4.54). The likelihood of developing OAB rose by 3.3 times for women who gave birth (OR 3.36, 95% CI 1.76–6.42). The presence of chronic diseases was associated with a 2.7-fold increase in the probability of developing OAB (OR 2.74, 95% CI 1.51–4.96). Conclusions: This study found that UI and OAB are common problems among female university students in Kazakhstan. The risk factors identified as childbirth and physical activity level emphasize the need to raise awareness of these conditions and their impact on quality of life. The results of the study can serve as a basis for the development of targeted prevention programs and support strategies for young women.

## 1. Introduction

Urinary incontinence (UI) is defined as the involuntary loss of urine, typically occurring in inappropriate settings, which leads to social and hygienic difficulties [1].

UI is a common problem among women, with a significant impact on quality of life and serious personal discomfort and social expenditures [2]. There are three main types of UI: stress UI, which involves urine leaks during physical activity, and overactive UI, which is characterized by a sudden and irresistible urge to urinate. Women who experience both of these symptoms are classified as having mixed UI [3].

The frequency of diagnosis of UI is higher in women due to the anatomical features of their genitourinary system. Although this condition can also occur in men, its prevalence is much lower, especially at a young age [4]. Factors contributing to the development of UI include urinary tract infections, genital prolapse, hormonal changes during menopause, and the consequences of complicated childbirth [5,6,7,8]. Age-related changes and comorbidities such as diabetes and cognitive disorders also play an important role in causing this problem [9,10]. Therefore, the relevance of UI requires a comprehensive approach to diagnosis and treatment, which is supported by numerous studies and proposed methods of correction [11].

Overactive bladder (OAB) is one of the most common conditions in the world, affecting about 100 million people and exceeding diabetes and peptic ulcer disease in frequency [12]. In Europe, about 17% of adults experience symptoms of OAB [13], and the prevalence of this condition is about 10.5 million people in Russia, of whom 36% suffer from urge UI [14]. This emphasizes how crucially important this problem is in different age groups.

The social significance of OAB is expressed in its impact on the quality of life of patients, affecting social, psychological, physical, and occupational aspects [15].

It is important to determine the frequency and risk factors for UI so that preventive measures could be developed. Symptoms of UI and OAB can be reduced or eliminated by lifestyle changes and conservative treatments [16,17,18].

Studies indicate that the prevalence of UI among women varies widely. Most studies concentrate on perimenopausal and postmenopausal women, suggesting that this issue is particularly significant among older women and those who have given birth [19,20]. However, some epidemiological studies also show that UI can affect younger women as well [21,22,23].

Previously, there has been no research conducted in Kazakhstan on the prevalence of UI and OAB among female students. Therefore, the aim of our study is to investigate the prevalence and potential risk factors of UI and OAB among female university students.

## 2. Materials and Methods

A cross-sectional study was conducted in the form of an online survey from 1 January 2024 to 31 December 2024.

A structured anonymous questionnaire was used. The study was conducted in accordance with the principles of the Helsinki Declaration of 1975, revised in 2024. All the participants provided anonymous informed consent to participate in the survey before proceeding to complete the questionnaire electronically. The study protocol was approved by the Local Bioethics Commission of Astana Medical University (No. 6 date of approval 3 October 2023).

The survey was conducted using a questionnaire that was sent by e-mail by the dean’s office staff of the relevant faculties to students of various specialties at the university. After clicking on the links, participants first read the title and description of the study, as well as information about their rights as participants. They then checked the appropriate boxes to confirm their consent to participate in the study before continuing with the survey. Additionally, the questionnaire included the researcher’s contact information so that participants could reach out in case a diagnosis was made.

After completing the questionnaire, the data were compiled into an Excel spreadsheet for analysis. All data collected from participants were handled anonymously and confidentially. Personal identifying information was securely protected and available only to a limited number of authorized individuals.

### 2.1. Study Size

The Epi Info program was used to calculate the sample with a confidence level of 95% and an error of 5%, as well as an expected frequency of 30% [24].

**Figure 1:**
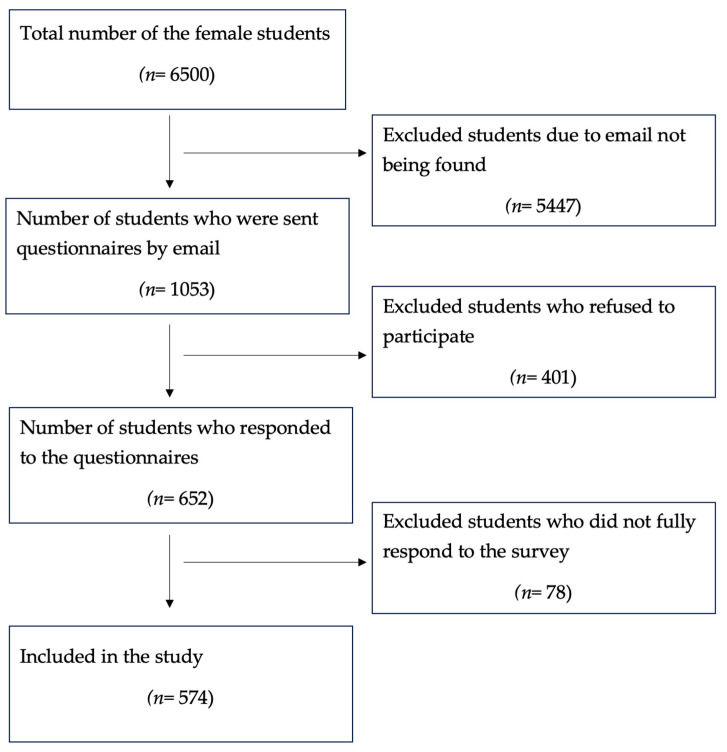
Flow chart of the selection process.

The sample selection process is shown in Figure 1. The total number of female students was 6500, of which 574 took part in the study.

### 2.2. Questionnaire Format

The first part of the questionnaire outlined the questions aimed at identifying risk factors for development of UI and OAB, such as age, height, weight, ethnicity, marital status, number of births, as well as smoking and alcohol habits and level of physical activity. In addition, questions also covered the other health conditions, including enuresis in childhood and chronic diseases. The variables related to chronic diseases, smoking habits, and alcohol consumption were defined based on data collected in the present.

In the second part, the ICIQ-UI-SF was used to determine the frequency, type, severity and associated factors of UI, and the OAB-V8 questionnaire was used to determine OAB. The questionnaires validated in the Russian language were used for the survey [24].

The ICIQ-UI-SF scale was developed by the International Incontinence Society to assess UI and its impact on quality of life. It demonstrates a satisfactory level of sensitivity, validity, and reliability. The scale consists of four items in addition to age and gender. The first two items track the frequency of UI and the volume of urine leaking, respectively. The third element assesses the extent to which the quality of daily life is impaired due to incontinence. The total ICIQ-UI-SF score is calculated by summing the scores of these three elements and ranges from 0 to 21, which provides a measure of severity and concern: 0 for no incontinence; 1–5 for mild; 6–12 for moderate; 13–18 for severe; and 21 for a very severe urine leakage [25]. The fourth element of the questionnaire helps to identify possible causes of incontinence and categorizes it as stress incontinence (occurring when coughing, sneezing or exercising), urge incontinence (occurring before going to the toilet), or mixed incontinence (a combination of stress and urge incontinence).

The method for estimating the prevalence of UI was to calculate the ratio of participants with UI to the total number of participants.

The OAB-V8 is an adapted questionnaire for assessing OAB symptom bother based on the OAB-q. It includes 8 items and assesses four core symptoms: urinary frequency, urgency, nocturia, and incontinence using a 6-point Likert scale. The total score is calculated as the sum of the answers to all questions and can range from 0 to 40. Respondents with a total OAB-V8 score ≥ 8 are considered to have OAB [26].

The prevalence estimation method was to calculate the ratio of participants with OAB to the total number of participants.

### 2.3. Statistical Analysis

StatTech v. 4.8.0 (Russia) was used for statistical analysis.

Categorical data were described with absolute and relative frequencies. We calculated 95% confidence intervals for proportions using the Clopper–Pearson method. Comparison of frequencies in the analysis of 2 by 2 contingency tables was performed using Pearson’s chi-square test (for expected values greater than 10). The probability of depression was also analyzed using logistic regression with 95% confidence intervals, conducting both unadjusted and adjusted analyses. Nagelkerke pseudo-R^2^ was used as a measure of the model performance. A predictive model was developed to determine the probability of UI and OAB based on independent variables such as BMI, history of childbirth, presence of chronic diseases, smoking habits, physical activity level, childhood enuresis, and daily fluid consumption. Differences were considered statistically significant at *p* < 0.05.

## 3. Results

### 3.1. Demographic Characteristics

A total of 574 participants were included in the study. The median age of female students was 22.00 years (IQR 19.00–24.00). The median height of the participants was 165 cm (IQR 160–168) and the median weight was 57 kg (IQR 52.00–65.00). The largest proportion of participants were undergraduate students—54.4%, followed by interns—34.5%, residents—7.3%, doctoral students—2.1%, and master’s students, which made up only 1.7%. More than half of the participants reported that they do not exercise on a regular basis. Among the bad habits, 9.3% of the participants were smokers and 8.4% consumed alcohol. An overview of the socio-demographic characteristics of the participants is presented in Table 1.

### 3.2. Prevalence of UI and Risk Factors

Table 2 presents an assessment of factors associated with the presence of UI comparing two groups: patients with incontinence and patients without incontinence. The median ICIQ-SF questionnaire score was 1.00 (IQR 1.00–2.50). Among the respondents, 72.5% (*n* = 416) stated that they did not experience UI, while 27.5% (*n* = 158) of the participants reported having this problem. Of these, 17.4% (*n* = 100) had mild incontinence, 9.4% (*n* = 54) had moderate incontinence, and only four participants (*n* = 4) suffered from severe UI.

Our study evaluated factors associated with UI by comparing two groups: Group 1—Yes UI patients and Group 2—No UI. The results presented in Table 2 show that the percentage of patients with a BMI greater than 25 kg/m^2^ in the group with UI is 1.5 times higher than in the group without UI. However, the study did identify factors directly associated with the presence of UI: childbirth and participation in physical exercise.

The conducted analysis of other factors, such as the presence of chronic diseases, smoking, and alcohol consumption, also showed no statistically significant differences between the groups. However, the study did identify factors directly associated with the risk of UI, namely childbirth and exercising.

In the group of patients with UI, the percentage of women who have given birth was 24.1%, and in the group without UI it was 12%, which demonstrates a statistically significant result (*p* = 0.012). In addition, comparison of physical activity between the two groups also had a statistically significant result (*p* = 0.027). For example, among patients surveyed with UI, 19% said they never exercised, compared with 8.7% in the group without UI.

Table 3 presents the results of the logistic regression analysis, which assessed the probability of UI in relation to various variables. For each variable, the odds ratios (OR) with 95% confidence intervals (CI) and *p*-values are provided for both unadjusted and adjusted models.

Overall, the results indicate that the history of childbirth is the only variable with a statistically significant association with the probability of UI, both in the unadjusted and adjusted models. In the final adjusted analysis, we found that the odds of UI increased 2.3 times for women who have given birth (OR 2.30, 95% CI 1.16–4.54). Other factors did not show a significant impact on the likelihood of UI in this sample.

### 3.3. Prevalence of OAB and Risk Factors

The results presented in Table 4 evaluate the risk factors associated with OAB, with patients categorized into two groups based on their questionnaire scores: those with OAB (scores exceeding eight points) and those without OAB (scores less than eight points).

Among the respondents, 89.9% (*n* = 516) stated that they did not experience OAB, whereas 10.1% (*n* = 58) reported having the condition.

Data analysis showed that childbirth (*p* = 0.001), presence of chronic diseases (*p* = 0.006), and level of physical activity (*p* = 0.021) had a statistically significant association with the presence of OAB. Among women who have given birth, the presence of OAB was higher, as was among patients with chronic diseases and a sedentary lifestyle. Other factors were not associated with the existence of OAB.

Table 5 presents the results of a logistic regression analysis assessing the likelihood of OAB in relation to various variables. The results indicate that a history of childbirth is statistically significantly associated with the probability of OAB in both unadjusted and adjusted models. In the final adjusted analysis, we found that the odds of developing OAB increased 3.3 times for women who have given birth (OR 3.36, 95% CI 1.76–6.42). Additionally, the presence of chronic diseases was associated with a 2.7-fold increase in the likelihood of developing OAB (OR 2.74, 95% CI 1.51–4.96). In the unadjusted analysis, a history of enuresis showed a statistically significant association. However, this association was not statistically significant in the adjusted models. Other factors did not have a substantial impact on the likelihood of OAB in this sample. During the logistic regression analysis, independent variables such as physical activity level and daily fluid consumption were not included due to a lack of data. At present, it is impossible to determine which specific categories of physical exercise and the amount of fluid intake may be associated with the presence of OAB.

## 4. Discussion

Our study was the first in Kazakhstan devoted to the prevalence of UI and OAB among female students. According to the data obtained, the prevalence of UI among female students was 27.5%. At the same time, 10.1% of respondents reported having an OAB.

UI is a common condition affecting women of all ages. Although the condition is not directly life-threatening, it has a negative impact on physical and mental health, significantly reducing women’s quality of life [27]. According to the literature, 24% to 45% of women experience UI. In the age group of 20 to 39 years, this figure ranges from 7% to 37%, indicating the presence of incontinence in various forms. Among women over the age of 60, approximately 9% to 39% report daily problems with UI [1].

According to literature data, the prevalence of UI among young women ranges from 20% to 30% [28]. In our study, this figure was 27.5%, which once again confirms the relevance of the problem in this age group.

The prevalence of UI varies across different age groups, typically being lower in young adults, peaking around menopause, and gradually increasing in those aged 60 to 80. Studies indicate significant rates of UI among women in European countries, with prevalence ranging from 23% to 44% [28]. In younger populations, a notable prevalence of UI 38.5% was found among women aged 25 to 45 in Spain [29], while 32% of female college students in Iceland reported experiencing UI, with many experiencing a negative impact on their quality of life [30]. Among non-pregnant women engaged in intense sports, UI prevalence was noted at 22.9%, particularly higher in those participating in high-intensity activities [31]. In adolescents and young women, a prevalence of 12.4% was observed, with increased BMI linked to higher rates in those under 19 [32]. Additional studies highlight that factors such as constipation and urinary retention significantly contribute to UI, with prevalence rates of 23.6% among college students in central China [33] and around 20.0% in Turkey, where a notable portion of the population also experiences OAB [34,35].

A systematic review of 53 studies involving 610,438 participants found a global prevalence of OAB at 20%, which has increased by 18.1% over the past 20 years. Higher rates were observed among overweight individuals and those aged 60 and older [36]. Another study reported that 5.2% of adult women in South Korea have OAB. Significant associations were identified between OAB and risk factors such as age, marital status, high BMI, smoking, prolonged sleep, as well as a history of hypertension, diabetes, hyperlipidemia, and stroke [37].

Factors contributing to an increased risk of developing UI include pregnancy, childbirth, diabetes, and high BMI [38].

In our study, data analysis showed that factors such as the history of childbirth and the level of physical activity are associated with UI, according to ICIQ-UI-SF questionnare.

In our study, the group with UI exhibited lower levels of physical activity, which were found to be associated with the condition. Concurrently, existing literature corroborates this finding, highlighting the relationship between decreased physical activity and the prevalence of UI in various populations [39].

Various studies have established that UI serves as a significant indicator of the increased prevalence of this condition following childbirth [40,41,42]. According to the results of our study, the percentage of women who have given birth was twice as high in the group with UI compared to the group without it. This finding suggests a significant association between childbirth and the occurrence of UI.

Several studies indicate that risk factors for developing OAB may include age, childbirth, obesity, certain chronic diseases, and lifestyle choices [43,44,45]. In our study, childbirth, the presence of chronic diseases, and the level of physical activity were also associated with OAB. In a previous study, the rates of OAB were significantly higher among female students with chronic diseases [46]. Additionally, prior research has shown that individuals who engage in regular physical activity are at a lower risk of developing OAB compared to those who are inactive [47,48]

### Limitations

In this study, risk factors associated with UI and OAB among female students were investigated. However, there are certain limitations that may affect the interpretation of the results. First, the limited sample size of 574 participants may not allow for generalization of the findings to a broader population, as all participants are students, which does not reflect the characteristics of other age groups and social categories. Second, the use of self-reported data may lead to underestimation or overestimation of the issue, as participants may not disclose information about their condition due to stigma or embarrassment. Additionally, the lack of long-term follow-up limits the ability to identify causal relationships between risk factors and UI. Some potential factors, such as genetic predisposition and stress levels, were not considered, which may impact the comprehensiveness of the analysis.

Despite identifying some statistically significant differences, not all comparisons reached statistical significance, highlighting the need for more in-depth analysis and an increased sample size to confirm the results.

These limitations should be taken into account when interpreting the results and formulating recommendations for further research in this area.

## 5. Conclusions

The study investigated the prevalence of UI and OAB among female university students, as well as identified associations with factors related to the presence of these conditions. The findings revealed that 27.5% of the female participants experienced UI problems, with mild incontinence occurring in 17.4% of participants while only four participants suffered from severe incontinence. The identified factors, such as childbirth and physical activity level, have a statistically significant association with the presence of UI. The prevalence of OAB among respondents was 10.1%. The identified factors, such as childbirth, the presence of chronic diseases, and levels of physical activity, were significantly associated with the occurrence of this condition.

The results of this study emphasize the need for enhanced preventative measures aimed at supporting women after childbirth and those with chronic illnesses. Further research is required to better understand the mechanisms contributing to the development of these conditions and to develop effective prevention and treatment strategies.

## Figures and Tables

**Table 1:** Sociodemographic characteristics of participants.

Characteristics	*n* = 574	Percentage
Age		
<19	126	22.0
19–23	280	48.8
>23	168	29.2
Educational program		
Bachelor	312	54.4
Internship	198	34.5
Master	10	1.7
Residency	42	7.3
Doctoral	12	2.1
BMI (kg/m^2^)		
<18.5	88	15.3
18.5–24.9	82	14.3
>25	404	70.4
History of childbirth		
Yes	88	15.3
No	486	84.7
Chronic diseases		
Yes	148	25.8
No	426	74.2
Smoking habit		
Electronic cigarettes	42	7.3
Yes, less than 10 cigarettes a day	10	1.7
Yes, less than 20 cigarettes a day	2	0.3
No, I don’t smoke	520	90.6
Alcohol consumption		
Yes	48	8.4
No	526	91.6
Physical exercise status		
Twice a week	134	23.3
Every day	48	8.4
Irregularly	326	56.8
Never	66	1.5
Childhood enuresis		
Yes	96	16.7
No	478	83.3
Daily fluid consumption		
<1500 mL	406	70.7
1500–3000 mL	160	27.9
>3000 mL	8	1.4

**Table 2:** Comparison of risk factors between groups with and without UI.

Characteristics	UI			*p*-Value
		Yes (%)	No (%)	
BMI (kg/m^2^)	<18.5	20 (12.7)	68 (16.4)	0.183
	18.5–24.9	106 (67.0)	298 (71.6)	
	>25	32 (20.3)	50 (12.0)	
History of childbirth	Yes	38 (24.1)	50 (12.0)	0.012
	No	120 (75.9)	366 (88.0)	
Chronic diseases	Yes	44 (27.8)	104 (25.0)	0.622
	No	114 (72.2)	312 (75.0)	
Smoking habit	Yes	16 (10.1)	38 (9.1)	0.822
	No	142 (89.9)	378 (90.9)	
Alcohol consumption	Yes	16 (10.1)	32 (7.7)	0.483
	No	142 (89.9)	384 (92.3)	
Physical exercise status	Twice a week	42 (26.6)	92 (22.1)	0.027
	Every day	16 (10.1)	32 (7.7)	
	Irregularly	70 (44.3)	256 (61.5)	
	Never	30 (19.0)	36 (8.7)	
Childhood enuresis	Yes	24 (15.2)	72 (17.3)	0.668
	No	134 (84.8)	344 (82.7)	
Daily fluid consumption	<1500 mL	106 (67.1)	300 (72.1)	0.257
	1500–3000 mL	52 (32.9)	108 (26.0)	
	>3000 mL	0 (0.0)	8 (1.9)	

**Table 3:** Unadjusted and adjusted odds ratios (95% CI) for association of factors with UI.

Variables	Unadjusted		Adjusted	
	OR (CI 95%)	*p*-Value	OR (CI 95%)	*p*-Value
BMI (kg/m^2^)	1.06 (0.99–1.14)	0.069	1.06 (0.98–1.14)	0.120
To have a history of childbirth	2.31 (1.19–4.50)	**0.013**	2.30 (1.16–4.54)	0.016
To have chronic diseases	1.15 (0.64–2.07)	0.622	1.14 (0.61–2.13)	0.661
To have smoking habit	1.12 (0.47–2.67)	0.797	0.98 (0.35–2.75)	0.977
Physical exercise status				
Every day	1.09 (0.40–2.95)	0.858	1.07 (0.38–3.05)	0.887
Twice a week	1.27 (0.70–2.31)	0.425	1.33 (0.71–2.52)	0.368
Irregularly	0.59 (0.31–1.13)	0.115	0.58 (0.30–1.15)	0.122
Never (reference category)				
To have a childhood enuresis	0.85 (0.42–1.74)	0.668	0.73 (0.33–1.59)	0.432
Daily fluid consumption				
<1500 mL	0.73 (0.41–1.28)	0.281	0.72 (0.39–1.31)	0.292
1500–3000 mL	1.39 (0.79–2.45)	0.242	1.40 (0.77–2.55)	0.267
>3000 mL (reference category)				

**Table 4:** Comparison of risk factors between groups with and without OAB.

Characteristics	OAB			*p*-Value
		Yes (%)	No (%)	
BMI (kg/m^2^)	<18.5	84 (16.3)	4 (6.9)	0.144
	18.5–24.9	354 (68.6)	50 (86.2)	
	>25	78 (15.1)	4 (6.9)	
History of childbirth	Yes	66 (12.8)	22 (37.9)	0.001
	No	450 (87.2)	36 (62.1)	
Chronic diseases	Yes	120 (23.3)	28 (48.3)	0.006
	No	396 (76.7)	30 (51.7)	
Smoking habit	Yes	46 (8.9)	8 (13.8)	0.333
	No	470 (91.1)	50 (86.2)	
Alcohol consumption	Yes	42 (8.1)	6 (10.3)	0.721
	No	474 (91.9)	52 (89.7)	
Physical exercise status	Twice a week	128 (24.8)	6 (10.3)	0.021
	Every day	44 (8.5)	4 (6.9)	
	Irregularly	294 (57.0)	32 (55.2)	
	Never	50 (9.7)	16 (27.6)	
Childhood enuresis	Yes	80 (15.5)	16 (27.6)	0.115
	No	436 (84.5)	42 (72.4)	
Daily fluid consumption	<1500 mL	362 (70.2)	44 (75.9)	0.694
	1500–3000 mL	146 (28.3)	14 (24.1)	
	>3000 mL	8 (1.6)	0 (0.0)	

**Table 5:** Unadjusted and adjusted odds ratios (95% CI) for association of factors with OAB.

Variables	Unadjusted		Adjusted	
	OR (CI 95%)	*p*-Value	OR (CI 95%)	*p*-Value
BMI (kg/m^2^)	1.03 (0.96–1.11)	0.385	1.02 (0.94–1.10)	0.603
To have a history of childbirth	4.14 (2.29–7.48)	<0.001	3.36 (1.76–6.42)	<0.001
To have chronic diseases	3.13 (1.80–5.46)	<0.001	2.74 (1.51–4.96)	0.001
To have smoking habit	1.72 (0.76–3.86)	0.188	1.84 (0.62–5.49)	0.269
To have a childhood enuresis	2.02 (1.08–3.78)	**0.026**	1.50 (0.74–3.02)	0.256

## Data Availability

Data are available upon reasonable request.

## Abbreviations

The following abbreviations are used in this manuscript:

UI	Urinary Incontinence
OAB	Overactive Bladder
ICIQ-UI-SF	International Consultation on Urinary Incontinence Questionnaire Short Form
OAB-V8	V8 Overactive Bladder Questionnaire
IQR	Interquartile Range
BMI	Body Mass Index
OR	Odds Ratio
CI	Confidence Interval

## References

[1] Abrams P., Cardozo L., Fall M., Griffiths D., Rosier P., Ulmsten U., Van Kerrebroeck P., Victor A., Wein A. (2003). Standardisation Sub-Committee of the International Continence Society. The standardisation of terminology in lower urinary tract function: Report from the standardisation sub-committee of the International Continence Society. Urology.

[2] Mota R.L. (2017). Female Urinary Incontinence and Sexuality. Int. Braz. J. Urol..

[3] Haylen B.T., de Ridder D., Freeman R.M., Swift S.E., Berghmans B., Lee J., Monga A., Petri E., Rizk D.E., Sand P.K. (2010). An International Urogynecol.ogical Association (IUGA)/International Continence Society (ICS) joint report on the terminology for female pelvic floor dysfunction. Int. Urogynecol. J..

[4] Aoki Y., Brown H.W., Brubaker L., Cornu J.N., Daly J.O., Cartwright R. (2017). Urinary Incontinence in Women. Nat. Rev. Dis. Primers.

[5] Mair T. (2022). Urinary Incontinence and Urinary Tract Infections. Vet. Clin. N. Am. Equine Pr..

[6] Tunn R., Baessler K., Knüpfer S., Hampel C. (2023). Urinary Incontinence and Pelvic Organ Prolapse in Women. Dtsch. Arztebl. Int..

[7] Kołodyńska G., Zalewski M., Rożek-Piechura K. (2019). Urinary Incontinence in Postmenopausal Women—Causes, Symptoms, Treatment. Prz. Menopauzalny.

[8] Hallock J.L., Handa V.L. (2016). The Epidemiology of Pelvic Floor Disorders and Childbirth: An Update. Obs. Gynecol. Clin. N. Am..

[9] Nazzal Z., Khatib B., Al-Quqa B., Abu-Taha L., Jaradat A. (2021). The Prevalence and Risk Factors of Urinary Incontinence among Women with Type 2 Diabetes in the North West Bank: A Cross-Sectional Study. Lancet.

[10] Kirschner-Hermanns R., Anding R. (2016). Characteristic Features of Urinary Incontinence—Diagnostic Investigation in Geriatric Patients. Aktuelle Urol..

[11] Jha S., Jeppson P.C., Dokmeci F., Marquini G.V., Sartori M.G.F., Moalli P., Malik S.A. (2024). Management of Mixed Urinary Incontinence: IUGA Committee Opinion. Int. Urogynecol. J..

[12] Leron E., Weintraub A.Y., Mastrolia S.A., Schwarzman P. (2018). Overactive Bladder Syndrome: Evaluation and Management. Curr. Urol..

[13] Temml C., Heidler S., Ponholzer A., Madersbacher S. (2005). Prevalence of the Overactive Bladder Syndrome by Applying the International Continence Society Definition. Eur. Urol..

[14] Kogan M.I., Zachoval R., Ozyurt C., Schäfer T., Christensen N. (2014). Epidemiology and Impact of Urinary Incontinence, Overactive Bladder, and Other Lower Urinary Tract Symptoms: Results of the EPIC Survey in Russia, Czech Republic, and Turkey. Curr. Med. Res. Opin..

[15] Sacco E., Tienforti D., D’Addessi A., Pinto F., Racioppi M., Totaro A., D’Agostino D., Marangi F., Bassi P. (2010). Social, Economic, and Health Utility Considerations in the Treatment of Overactive Bladder. Open Access J. Urol..

[16] Imamura M., Williams K., Wells M., McGrother C. (2015). Lifestyle Interventions for the Treatment of Urinary Incontinence in Adults. Cochrane Database Syst. Rev..

[17] Nightingale G. (2020). Management of Urinary Incontinence. Post. Reprod. Health.

[18] Patel U.J., Godecker A.L., Giles D.L., Brown H.W. (2022). Updated Prevalence of Urinary Incontinence in Women: 2015-2018 National Population-Based Survey Data. Female Pelvic Med. Reconstr. Surg..

[19] Minassian V.A., Stewart W.F., Wood G.C. (2008). Urinary Incontinence in Women: Variation in Prevalence Estimates and Risk Factors. Obs. Gynecol..

[20] Joseph C., Srivastava K., Ochuba O., Ruo S., Cureus T., Sandhu J.K., Waqar A., Jain A., Poudel S. (2021). Stress Urinary Incontinence Among Young Nulliparous Female Athletes. Cureus.

[21] Tayla J., Lamerton M.A., Gregore I.M., Wendy J.B. (2020). Urinary Incontinence in Young Women: Risk Factors, Management Strategies, Help-Seeking Behavior, and Perceptions About Bladder Control. Neurourol. Urodyn..

[22] Robinson D., Cardozowan L. (2014). Urinary Incontinence in the Young Woman: Treatment Plans and Options Available. Women’s Health.

[23] Nitti V.W. (2001). The Prelevalence of Urinary Incontinence. Rev. Urol..

[24] Russian Version of Questionnaires for Life Quality Assessment in Patients with Pelvic Organ Prolapse and Stress Urinary Incontinence. Experimental and Clinical Urology. https://ecuro.ru/en/article/russian-version-questionnaires-life-quality-assessment-patients-pelvic-organ-prolapse-and-st.

[25] Klovning A., Avery K., Sandvik H., Hunskaar S. (2009). Comparison of two questionnaires for assessing the severity of urinary incontinence: The ICIQ-UI SF versus the incontinence severity index. Neurourol. Urodyn..

[26] Acquadro C., Kopp Z., Coyne K.S., Corcos J., Tubaro A., Choo M.-S., Oh S.J. (2006). Translating Overactive Bladder Questionnaires in 14 Languages. Urology.

[27] Fontaine C., Papworth E., Pascoe J., Hashim H. (2021). Update on the Management of Overactive Bladder. Ther. Adv. Urol..

[28] Hunskaar S., Lose G., Sykes D., Voss S. (2004). The Prevalence of Urinary Incontinence in Women in Four European Countries. BJU Int..

[29] Cabello-Díaz B., Tirado-Morata C., Garrido-Elustondo S. (2025). Prevalence of urinary incontinence in young and nulligravid women. Aten. Primaria.

[30] Geirsson G., Hansen B., Hermannsdóttir K. (2003). Prevalence of urinary incontinence among young female college students. Laeknabladid.

[31] Alves J.O., Luz S.T.D., Brandão S., Da Luz C.M., Jorge R.N., Da Roza T. (2017). Urinary Incontinence in Physically Active Young Women: Prevalence and Related Factors. Int. J. Sports Med..

[32] Bardino M., Di Martino M., Ricci E., Parazzini F. (2015). Frequency and Determinants of Urinary Incontinence in Adolescent and Young Nulliparous Women. J. Pediatr. Adolesc. Gynecol..

[33] Zhou F., Xue K., Liu Y., Zhuo L., Tu S., Palmer M.H. (2020). Toileting Behaviors and Factors Associated with Urinary Incontinence in College-Aged Female Students in China. Int. Urogynecol. J..

[34] Karasu A.F.G., Cetin C., Pasin Ö., Karacabay M., Tanoglu F.B., Ilhan G. (2023). Prevalence of Urinary Incontinence and Anal Incontinence: An Internet-Based Cross-Sectional Study of Female Turkish University Students. Int. Urogynecol. J..

[35] Ural Ü.M., Gücük S., Ekici A., Topçuoğlu A. (2021). Urinary Incontinence in Female University Students. Int. Urogynecol. J..

[36] Zhang L., Cai N., Mo L., Tian X., Liu H., Yu B. (2025). Global Prevalence of Overactive Bladder: A Systematic Review and Meta-Analysis. Int. Urogynecol. J..

[37] Kim S.Y., Bang W., Choi H.G. (2017). Analysis of the prevalence of and factors associated with overactive bladder in adult Korean women. PLoS ONE.

[38] Abuorouq S., Al-Zubi M., Al-Ali A.M., Aloqaily L.H., Talafha M.A., Migdadi A.M., Serhan H.A. (2024). The Prevalence of Probable Overactive Bladder and Associated Risk Factors among Medical Students in Jordan: A Cross-Sectional Study. BMC Urol..

[39] Sun C., Duan Z. (2024). Joint Effect of Physical Activity and Sedentary Behavior with the Female Urinary Incontinence: An Analysis of NHANES 2011–2016. Urol. Int..

[40] Hvidman L., Foldspang A., Mommsen S., Nielsen J.B. (2003). Postpartum urinary incontinence. Acta Obs. Gynecol. Scand..

[41] Peyrat L., Haillot O., Bruyere F., Boutin J.M., Bertrand P., Lanson Y. (2002). Prévalence et facteurs de risque de l’incontinence urinaire chez la femme jeune Prevalence and risk factors of urinary incontinence in young women. Prog. Urol..

[42] Zizzi P.T., Trevisan K.F., Leister N., Cruz C.D., Riesco M.L. (2017). Women’s pelvic floor muscle strength and urinary and anal incontinence after childbirth: A cross-sectional study. Rev. Esc. Enferm. USP.

[43] Coyne K.S., Sexton C.C., Thompson C.L., Milsom I., Irwin D., Kopp Z.S., Chapple C.R., Kaplan S., Tubaro A., Aiyer L.P. (2009). The Prevalence of Lower Urinary Tract Symptoms (LUTS) in the USA, the UK and Sweden: Results from the Epidemiology of LUTS (EpiLUTS) Study. BJU Int..

[44] Zhu J., Hu X., Dong X., Li L. (2019). Associations Between Risk Factors and Overactive Bladder: A Meta-analysis. Female Pelvic Med. Reconstr. Surg..

[45] Ahmad S.M., Aznal S.S., Tham S.W. (2015). Prevalence of Overactive Bladder Syndrome (OABS) Among Women with Gynaecological Problems and Its Risk Factors in a Tertiary Hospital, Negeri Sembilan, Malaysia: Implication for Primary Healthcare Providers. Malays. Fam. Physician.

[46] Shawahna R., Hijaz H., Jallad K., Abushamma M., Sawafta M. (2021). Prevalence of overactive bladder symptoms and their impact on health-related quality of life of medical and dentistry students: A multicenter cross-sectional study. BMC Urol..

[47] Coyne K.S., Sexton C.C., Clemens J.Q., Thompson C.L., Chen C.I., Bavendam T., Dmochowski R. (2013). The impact of OAB on physical activity in the United States: Results from OAB-POLL. Urology.

[48] Wu T., Xu B. (2025). The relationship between physical activity and overactive bladder among American adults: A cross-sectional study from NHANES 2007–2018. Sci. Rep..

